# Light-responsive transcription factor PpWRKY44 induces anthocyanin accumulation by regulating *PpMYB10* expression in pear

**DOI:** 10.1093/hr/uhac199

**Published:** 2022-09-06

**Authors:** Ahmed Alabd, Mudassar Ahmad, Xiao Zhang, Yuhao Gao, Lin Peng, Lu Zhang, Junbei Ni, Songling Bai, Yuanwen Teng

**Affiliations:** College of Agriculture and Biotechnology, Zhejiang University, Hangzhou, Zhejiang 310058, China; Department of Pomology, Faculty of Agriculture, Alexandria University, Alexandria 21545, Egypt; College of Agriculture and Biotechnology, Zhejiang University, Hangzhou, Zhejiang 310058, China; College of Agriculture and Biotechnology, Zhejiang University, Hangzhou, Zhejiang 310058, China; College of Agriculture and Biotechnology, Zhejiang University, Hangzhou, Zhejiang 310058, China; College of Agriculture and Biotechnology, Zhejiang University, Hangzhou, Zhejiang 310058, China; College of Agriculture and Biotechnology, Zhejiang University, Hangzhou, Zhejiang 310058, China; College of Agriculture and Biotechnology, Zhejiang University, Hangzhou, Zhejiang 310058, China; College of Agriculture and Biotechnology, Zhejiang University, Hangzhou, Zhejiang 310058, China; College of Agriculture and Biotechnology, Zhejiang University, Hangzhou, Zhejiang 310058, China; Hainan Institute of Zhejiang University, Sanya, Hainan 572025, China

## Abstract

Anthocyanins are a valuable source of antioxidants in the human diet and contribute to fruit coloration. In red-skinned pears, anthocyanin biosynthesis can be induced by light, in which the MYB–bHLH–WDR complex plays a critically important role in transcriptional regulation. However, knowledge of WRKY-mediated transcriptional regulation of light-induced anthocyanin biosynthesis is scarce in red pears. This work identified and functionally characterized a light-inducing WRKY transcription factor, PpWRKY44, in pear. Functional analysis based on overexpressed pear calli showed that PpWRKY44 promoted anthocyanin accumulation. Also, transiently overexpressed *PpWRKY44* in pear leaves and fruit peels significantly enhanced the accumulation of anthocyanin, whereas silencing *PpWRKY44* in pear fruit peels impaired induction of the accumulation of anthocyanin by light. By chromatin immunoprecipitation and electrophoretic mobility shift assay coupled to a quantitative polymerase chain reaction, we found that PpWRKY44 bound *in vivo* and *in vitro* to the *PpMYB10* promoter, revealing it as a direct downstream target gene. Moreover, *PpWRKY44* was activated by PpBBX18, a light signal transduction pathway component. Our results explained the mechanism mediating the impacts of PpWRKY44 on the transcriptional regulation of anthocyanin accumulation, with potential implications for fine-tuning the fruit peel coloration triggered by light in red pears.

## Introduction

Pear (*Pyrus* L*.*), as one of the fruit crops produced in temperate regions, is economically significant thanks to the health benefits accompanying its edible fruit. In recent years, red-skinned pears have emerged as a fruit popular among consumers. The red pear fruit skin is attributable to anthocyanin accumulation [1]. Anthocyanins are an important secondary metabolite belonging to a class of phenylpropanoid compounds called flavonoids [2]. Their beneficial effects on humans are related to their health-promoting antioxidative properties, which can protect against cardiovascular disorders and degenerative diseases [3, 4]. In plants, anthocyanins perform various functions, such as fertility, defensive responses against plant pathogens, protection against UV light, and antioxidant activity [5, 6]. Thus, the regulatory systems controlling anthocyanin biosynthesis are the main focus of research.

Anthocyanin biosynthesis occurs within the phenylpropanoid pathway as part of the flavonoid branch. It is executed via a series of structural genes and is catalyzed by numerous well-documented enzymes. These enzymes include phenylalanine ammonia-lyase (PAL), chalcone synthase (CHS), chalcone isomerase (CHI), flavanone 3-hydroxylase (F3H), dihydroflavonol-reductase (DFR), anthocyanidin synthase (ANS), and UDP-glucose:flavonoid 3-glucosyltransferase (UFGT) [5, 7]. The transcriptional regulation of the genes encoding these enzymes is tuned by a conserved MYB–bHLH–WDR (MBW) complex, which comprises MYB transcription factors (TFs), basic helix–loop–helix (bHLH) TFs, and WD-repeat proteins (e.g. WD40) [7, 8]. R2R3-MYB TFs are among the best-characterized TFs as critical transcriptional regulators of anthocyanin structural genes [9]. The knockdown of *FvMYB10* resulted in the production of white strawberry fruit [10]. In apple, three MYB genes, *MdMYB10*, *MdMYB1*, and *MdMYBA*, which are closely related homologs of *Arabidopsis AtMYB75*/*PAP1* and *AtMYB90*/*PAP2*, encode TFs that directly trigger the transcriptional activation of anthocyanin structural genes [11–13]. In pear, both PpMYB10 [14] and PpMYB114 [15] are positively correlated with anthocyanin accumulation by directly acting upstream of anthocyanin structural genes. MYB-mediated regulation of anthocyanin accumulation at the transcriptional and post-translational levels depends on environmental stimuli, including low temperatures, water, salt, and light [16–18].

Light, an important environmental signal, strongly affects anthocyanin biosynthesis in several plant species [12, 19]. Light signals can be sensed by receptors and converted into physiological responses (e.g. anthocyanin biosynthesis) via various signal transduction pathways, in which MYB TFs play a critically essential role. In petunia (*Petunia hybrida*), anthocyanin accumulation in vegetative organs induced by light is tightly correlated to the expression levels of genes encoding anthocyanin-associated MYB TFs [16]. ELONGATED HYPOCOTYL 5 (HY5), the central regulator of the signaling transduction pathway responsive to light, increases anthocyanin accumulation due to the direct regulation of anthocyanin-associated genes (*MYB* and structural genes) in several plant species [20, 21]. Additionally, our previous reports showed that two B-box proteins, PpBBX16 and PpBBX18, are light-dependent and can function together with PpHY5 to mediate anthocyanin accumulation in pear. Both proteins require PpHY5 to induce the expression of the anthocyanin biosynthesis regulatory gene *PpMYB10* in pear [22, 23]. Other TFs, such as NAC, ERF, and WRKY, also affect transcriptional regulation of light-dependent anthocyanin biosynthesis in various fruit species [9] by functioning alone or as part of multiprotein complexes.

The WRKY TF is one of the main TFs in plants. It has at least one conserved 60 amino acid domain, called the WRKY domain, that comprises a highly conserved polypeptide (WRKYGQK) and a zinc finger motif at its N- and C-terminus, respectively [24]. The WRKY TFs are grouped into three subfamilies (Groups I, II, and III) based on the WRKY and zinc finger motif types. The Group I members are characterized by two WRKY domains with a zinc finger motif (C_2_H_2_-type). In contrast, Group II and III members contain only one WRKY domain with zinc finger motifs (C_2_HC- and C_2_H_2_-type). Moreover, Group II members may be divided into subgroups IIa, IIb, IIc, IId, and IIe based on their conserved motifs [24] All WRKY proteins play regulatory functions by binding to the DNA sequence (C/T)TGAC(T/C), called W-box elements, in the promoter region of their target genes [25]. Earlier studies showed that WRKY proteins serve as important regulators in many developmental and physiological processes, including leaf development [26], root growth [27, 28], seed development [29, 30], and senescence [26, 31, 32], and in plant responses to biotic and abiotic stresses [33, 34]. There are indications that members of these protein groups are also involved in secondary metabolism in plants. For example, GaWRKY1 helps regulate sesquiterpene biosynthesis in cotton by modulating the expression of *CAD1-A* [35]. In grape, VvWRKY26 induces the accumulation of flavonoids by targeting the structural genes of the flavonoid biosynthesis pathway [36]. There are a few documents describing the role of these proteins related to light-mediated anthocyanin biosynthesis. A recent study determined that BnWRKY41-1 controls anthocyanin accumulation, similar to AtWRKY41 in *Arabidopsis* rosette leaves in the presence of light [37]. In the presence of light, apple MdWRKY41 modulates anthocyanin accumulation by negatively regulating the transcriptions of *MdUFGT*, *MdANR*, and *MdMYB12* [38]. In contrast, apple MdWRKY11 activates *MdMYB10*-promoted anthocyanin accumulation [39]. Furthermore, in response to light, the MdWRKY1–MdLNC499–MdERF109 complex enhances anthocyanin accumulation by targeting the structural genes in the early stages of fruit coloration of apple. Briefly, light activates the expression of *MdWRKY1*, which leads to the upregulated transcription of *MdLNC499* and the formation of the MdERF109 protein, which increases the induction of the transcription of anthocyanin structural genes in apple fruit [40]. However, whether WRKY TFs are implicated in light-induced anthocyanin accumulation is less documented and data from red pears are scarce. In this study, a light-responsive Group-I WRKY TF (PpWRKY44) in ‘Hongzaosu’ pear fruit was identified. Our analyses clarified that PpWRKY44 positively regulates anthocyanin biosynthesis via the transcriptional regulation of *PpMYB10*. Additionally, based on the observed high luciferase activity and β-galactosidase (GUS) staining, PpBBX18 likely activates the *PpWRKY44* promoter. We determined that WRKY TFs are regulators of light-induced anthocyanin accumulation. Collectively, the findings of this study have further clarified the effects of WRKY-mediated transcriptional regulation of anthocyanin-related genes induced by light.

## Results

### Identification of candidate gene *PpWRKY44* and analysis of its expression in pear fruit and calli under light

**Figure 1:**
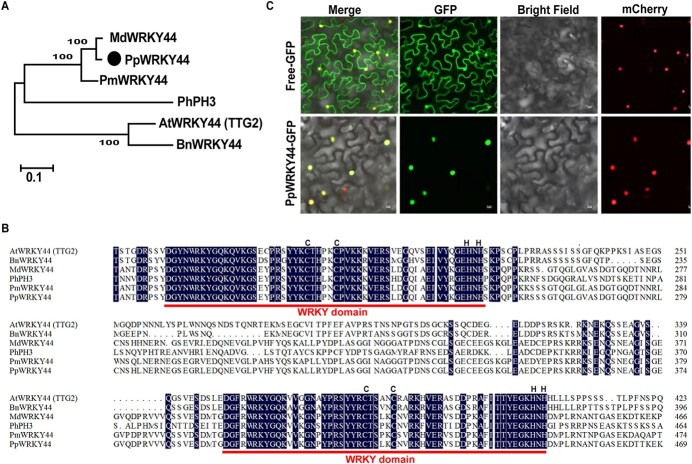
Sequence characteristics and subcellular localization of PpWRKY44. (A) Phylogenetic relationship of PpWRKY44 and other WRKYs from other species conducted on the basis of protein sequences. PpWRKY44 is marked by a black circle. (B) Sequence alignment of PpWRKY44 and other WRKY transcription factors. *Pp*, *Pyrus pyrifolia*; *Md*, *Malus domestica* (*MdWRKY44: XP_008387690.2*); *Pm*, *Prunus mume* (*PmWRKY44: XP_008242029.1*); Bn, *Brassica napus* (*BnWRKY44: XP_022557932.1*); *At*, *Arabidopsis thaliana* (*AtTTG2: NP_181263.2*); *Ph*, *Petunia hybrida* (*PhPh3: AMR43368*). Red lines represent the conserved WRKY amino acid domains, whereas black letters represent zinc finger motifs. (C) Nuclear localization of PpWRKY44 in tobacco leave cells. Scale bars = 10 μm.

Previous studies in pear have shown that substantial induction of anthocyanin biosynthesis can be achieved by light [41]. To identify candidate regulators belonging to WRKY TFs that may be involved in this process, we analyzed our previous transcriptomic data on pear calli exposed to light to induce anthocyanin biosynthesis [23, 41]. The candidate light-inducible WRKY gene was identified, and its expression was upregulated in calli after 2 days of light treatment (Supplementary Data Fig. S1). Phylogenetic analysis revealed that the candidate WRKY gene is closely related to *Arabidopsis* AtWRKY44/TTG2, which is a Group-I WRKY TF that regulates proanthocyanidin synthesis in the seed coat [42] (Supplementary Data Fig. S2). Thus, we named this pear WRKY TF PpWRKY44. A multiple protein sequence alignment indicated that PpWRKY44 contains C_2_H_2_-type zinc finger motifs and two WRKY domains, which are conserved in the WRKY44 proteins of other species (Fig. 1A and B). To determine the subcellular localization of PpWRKY44, we fused the *PpWRKY44* coding sequence with the green fluorescent protein (GFP)-encoding gene and transiently expressed the fusion construct in *Nicotiana benthamiana* leaves. A fluorescence examination based on GFP detection revealed that PpWRKY44–GFP is localized entirely to the nuclei. In contrast, GFP alone was found throughout the cell (Fig. 1C), demonstrating that PpWRKY44 is a nuclear protein.

**Figure 2:**
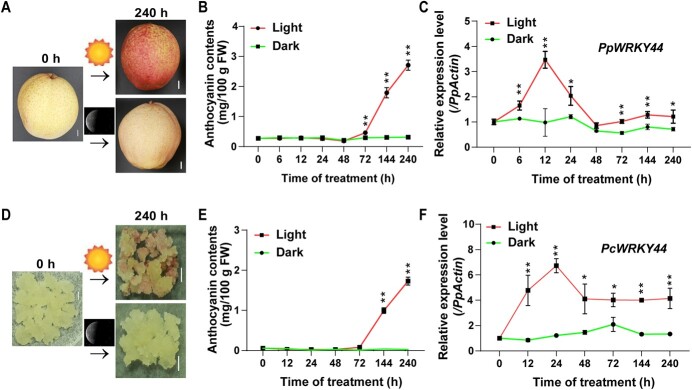
Light-responsive *PpWRKY44* expression pattern. (A) Light-induced phenotypes of ‘Hongzaosu’ pear fruits. Fruits at 0 and 240 hours are shown. Scale bars = 1 cm. (B) Content of anthocyanin for each sample time-point in fruit peel. (C) Expression patterns of *PpWRKY44* at each sample time-point during treatment. (D) Light-induced phenotypes of pear calli. Pear calli at 0 and 240 hours are shown. Scale bars = 1 cm. (E) Content of anthocyanin in pear calli at each sample time-point. (F) *PcWRKY44* expression patterns at each sample time-point. Error bars represent the standard deviation of three biological replicates. The expression level at 0 hours was used as the reference. ^*^P < .05, ^**^P < .01 (two-tailed Student’s *t*-test).

To analyze the *PpWRKY44* expression pattern in response to light, ‘Hongzaosu’ pear fruits were subjected to a 10-day light treatment. As expected, upon visual inspection of the pear tissue types, the light treatment displayed a strong red coloring that was not observed in the dark treatment (Fig. 2). In brief, anthocyanins started to accumulate after 48 hours in light-treated pear fruit peels. The content of anthocyanin subsequently continued increasing for the duration of the treatment. In contrast, accumulated anthocyanins were scarce in the dark-treated fruit (Fig. 2A and B). The RT–qPCR analysis of the effects of the light treatment revealed that the *PpWRKY44* expression level increased after 6 hours and peaked after 12 hours. Compared with its expression under light, *PpWRKY44* was expressed at lower levels in darkness (Fig. 2C). Furthermore, most anthocyanin-related genes (*PpBBX18*, *PpMYB10*, *PpMYB114*, *PpCHI, PpCHS*, *PpF3H*, *PpDFR*, *PpUFGT*, and *PpANS*) were expressed similarly to *PpWRKY44* following light and dark treatments (Supplementary Data Fig. S3). Considering that pear calli would be used as the plant material for subsequent experiments, we analyzed *PcWRKY44* expression in light-treated pear calli over a 10-day period. The data indicated that *PcWRKY44* expression increased after 12 hours and peaked after 24 hours, increasing ~6-fold, and the calli began to accumulate anthocyanins after 72 hours (Fig. 2D–F). Induction of the expression of PpWRKY44 by light treatment followed by increasing anthocyanin accumulation indicates that PpWRKY44 is likely a light-responsive regulator of anthocyanin biosynthesis in pear.

### PpWRKY44 promotes anthocyanin biosynthesis

**Figure 3:**
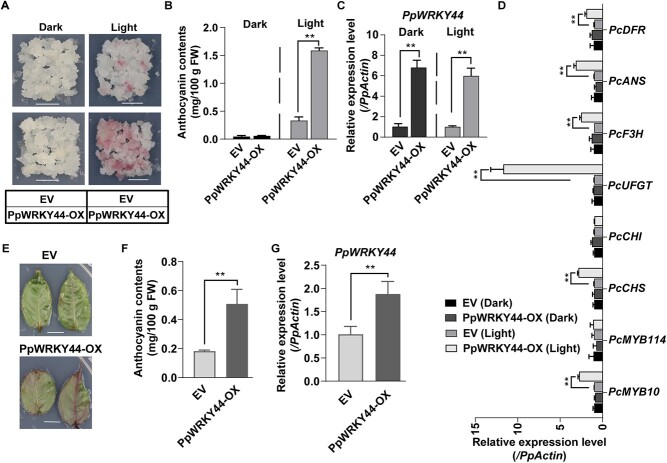
PpWRKY44 functional assay through its overexpression in pear calli and pear leaves. (A) Phenotypes of EV and *PpWRKY44*-OX calli after a 6-day light treatment. Scale bars = 1 cm. (B) Content of anthocyanin in EV and *PpWRKY44*-OX calli. (C) *PpWRKY44* expression level in EV and *PpWRKY44*-OX calli. (D) Relative transcript levels of *PcMYB10*, *PcMYB114*, *PcCHS*, *PcCHI*, *PcDFR*, *PpF3H*, and *PpANS* in EV and *PpWRKY44*-OX calli. (E) Phenotypes of leaves transiently transformed with *PpWRKY44*-OX or EV and (F) their anthocyanin contents after 3-day light treatment. (G) *PpWRKY44* expression level in transgenic pear leaves. Error bars represent the standard deviation of three biological replicates. The expression level of EV was used as the reference. ^**^P < .01 (two-tailed Student’s *t*-test).

To explore the regulatory effects of PpWRKY44 on light-induced anthocyanin accumulation, we developed *Pyrus communis* ‘Clapp’s Favorite’ transgenic calli overexpressing *PpWRKY44* (*PpWRKY44*-OX) via *Agrobacterium tumefaciens*-mediated transformation. The results of the RT–PCR and RT–qPCR analyses confirmed the presence of *PpWRKY44* in the transgenic calli (Fig. 3C; Supplementary Data Fig. S4). We subsequently investigated whether PpWRKY44 mediates anthocyanin biosynthesis in response to light. Specifically, soft and fast-growing *PpWRKY44*-OX and control calli containing the empty vector (EV) were treated with continuous light for 6 days. Upon visual inspection of the overexpressing *PpWRKY44*, the red color appeared to be increased in various tissue types of pear (Fig. 3). Anthocyanins accumulated considerably more in the *PpWRKY44*-OX calli than in the EV calli. In contrast, anthocyanins did not accumulate in calli incubated in darkness (Fig. 3A and B). The overexpression of *PpWRKY44* also dramatically led to an increase in *PcMYB10* expression, but not in *PcMYB114* expression (Fig. 3D). Besides, the anthocyanin biosynthetic pathway genes, including *PcCHI*, *PcCHS*, *PcF3H PcUFGT*, *PcANS*, and *PcDFR*, were also significantly upregulated in *PpWRKY44*-OX compared with EV in response to light. In contrast, when compared with EV, no significant differences were observed in expressions of anthocyanin biosynthetic pathway genes in *PpWRKY44*-OX under dark treatment (Fig. 3D). The PpWRKY44 function associated with anthocyanin biosynthesis regulation was validated by analyzing pear leaves transiently overexpressing *PpWRKY44*-OX or EV (Fig. 3E–G). Consistent with the examination results of pear calli, *PpWRKY44*-OX pear leaves accumulated substantially more anthocyanins than the pear leaves containing the EV (Fig. 3F).

**Figure 4:**
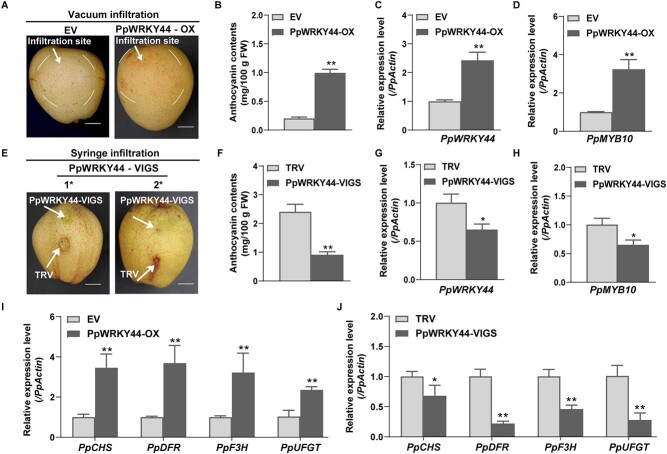
Transient expressions and silencing of *PpWRKY44* in pear fruit. (A) ‘Meirensu’ fruit anthocyanin accumulation in the transient overexpression of *PpWRKY44* after a 5-day light treatment. Scale bars = 1 cm. (B) Anthocyanin contents around infiltrated sites of fruit peel transiently overexpressing *PpWRKY44*. (C) Relative *PpWRKY44* transcript level in fruit transiently overexpressing *PpWRKY44*. (D) Relative *PpMYB10* transcript level in fruit transiently overexpressing *PpWRKY44*. (E) Transient silencing of *PpWRKY44* reduced the accumulation of anthocyanin in mature ‘Hongzaosu’ fruit after a 7-day light treatment. Scale bars = 1 cm. (F) Anthocyanin contents around the infiltrated sites of pear fruit in which *PpWRKY44* was transiently silenced. (G) *PpWRKY44* expression in pear fruit in which *PpWRKY44* was transiently silenced. (H) Relative *PpMYB10* transcript level in pear fruit in which *PpWRKY44* was transiently silenced. (I) Relative transcript levels of *PpCHS*, *PpDFR*, *PpF3H*, and *PpUFGT* genes in pear fruit transiently overexpressing *PpWRKY44*. (J) Relative transcript levels of *PpCHS*, *PpDFR*, *PpF3H*, and *PpUFGT* genes in pear fruit in which *PpWRKY44* was transiently silenced. Error bars represent the standard deviation of three biological replicates. The expression level of EV was used as the reference. ^*^P < .05, ^**^P < .01 (two-tailed Student’s *t*-test).

To further verify that PpWRKY44 modulates anthocyanin biosynthesis, the *PpWRKY44*-OX vector was vacuum-infiltratedinto ‘Meirensu’ pear fruit, whereas a virus-induced gene silencing (VIGS) vector (TRV2-*PpWRKY44*) was injected into ‘Hongzaosu’ pear fruit. Red coloration was detected around the infiltration site after 5 days of light treatment, but only for the *PpWRKY44*-OX fruit (Fig. 4A), which was in accordance with the anthocyanin content (Fig. 4B). Regarding the VIGS analysis, compared with the effectsof EV after a 7-day light treatment, the silencing of *PpWRKY44* had a suppressive effect on coloration and decreased the anthocyanin content around the injection site (Fig. 4E, F). Relative to the control, *PpWRKY44*-OX fruit displayed a significantly higher level of *PpMYB10* expression, while the *PpMYB10* expression level was significantly lower in TRV2-*PpWRKY44* fruit (Fig. 4D and H). Furthermore, a comparison with control fruit carrying the EV revealed that the *PpCHS*, *PpF3H*, *PpDFR*, and *PpUFGT* expression levels increased in the *PpWRKY44*-OX fruit but decreased in the TRV2-*PpWRKY44* fruit (Fig 4I and J). These results demonstrated that PpWRKY44 acts upstream of the regulation of *PpMYB10* transcription for light-induced anthocyanin biosynthesis in red pear fruit.

### PpWRKY44 activates the *PpMYB10* promoter

**Figure 5:**
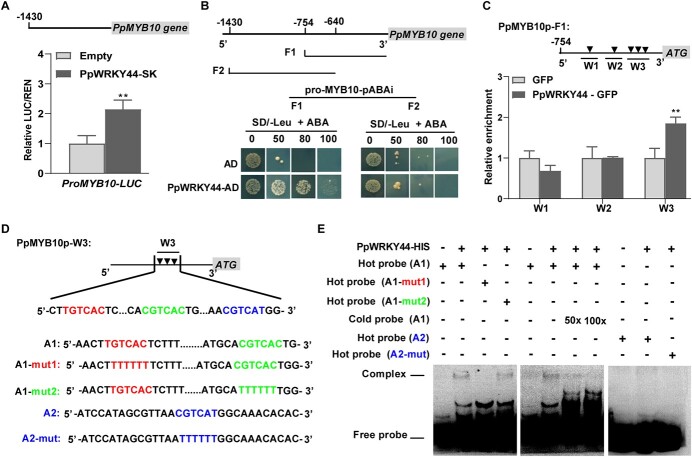
PpWRKY44 protein binds to the promoter of *PpMYB10*. (A) PpWRKY44 induced *PpMYB10* transcription in dual-luciferase assays. (B) Schematic diagram of *PpMYB10* promoter fragments F1 (start codon to −754 bp) and F2 (−640 to −1430 bp) and the interactions between PpWRKY44 and these fragments in yeast cells. (C) ChIP–qPCR assays showed that PpWRKY44 protein bound to the *PpMYB10* promoter. Chromatins from GFP and PpWRKY44–GFP pear calli were immunoprecipitated with or without a GFP antibody. Three regions (W1, W2, and W3) were analyzed by RT–qPCR. Enrichment of GFP was set to 1. (D) Schematic diagram of *PpMYB10* promoter fragment W3 used for the EMSAs. (E) EMSA results revealed the binding of PpWRKY44 protein to the W-box (TGTCAC) within the sequence A1 of the *PpMYB10* promoter. Error bars represent the standard deviation of three biological replicates. ^**^P < .01 (two-tailed Student’s *t*-test).

We recently confirmed that PpMYB10, a key regulator of light-induced anthocyanin biosynthesis, can bind to most anthocyanin structural gene promoters [41]. Because PpWRKY44 can induce the expression of *PpMYB10*, we imagined that PpWRKY44 protein can bind to the *PpMYB10* promoter to regulate transcription (Figs 3D and D). Hence, we analyzed the *PpMYB10* promoter region and identified many W-box elements, potential binding sites for WRKY TFs. A transient dual-luciferase assay revealed an increase in *PpMYB10* promoter activity in the presence of PpWRKY44 (Fig. 5A). Similarly, PpWRKY44 activated the promoters of most of the anthocyanin structural genes (Supplementary Data Fig. S5). For the yeast one-hybrid (Y1H) assays, we first constructed *PpMYB10* promoter fragments F1 (start codon to −754 bp) and F2 (−640 to −1430 bp). The two fragments were fused into the pAbAi vector (Fig. 5B). In the Y1H assays, PpWRKY44 was able to bind to F1 but not to F2, suggesting that PpWRKY44 can only bind to the W-box elements in F1 (Fig. 5B). The fragment F1 was further divided into three fragments (W1, W2, and W3) containing W-box elements for chromatin immunoprecipitation (ChIP)–qPCR assays. Specifically, we used the PpWRKY44–GFP transgenic calli and performed the ChIP–qPCR analysis using anti-GFP antibodies. The results confirmed that PpWRKY44 enriched fragment W3, irrespective of fragments W1 and W2 (Fig. 5C), indicating that PpWRKY44 recognizes fragment W3 in the *PpMYB10* promoter. Next, we examined fragment W3 for the presence of W-box elements and their reverse-complemented sequences, which resulted in the detection of three W-box elements (TGTCAC, CGTCAC, and CGTCAT) (Fig. 5D). Electrophoretic mobility shift assay (EMSA)s, which were performed using the recombinant PpWRKY44-His fusion protein, indicated that PpWRKY44 was able to bind to the probe of sequence A1 and cause a mobility shift, but it failed to bind to the probe of sequence A2 (Fig. 5E). Moreover, PpWRKY44 was still able to bind to the probe of sequence A1 when CGTCAC was mutated to TTTTTT, but not when TGTCAC was mutated to TTTTTT (Fig. 5D), implying that PpWRKY44 could bind directly to the W-box (TGTCAC) within the sequence A1 of the *PpMYB10* promoter. Additionally, an increase in the amount of the unlabeled probe of sequence A1 resulted in a decrease in the ability of PpWRKY44 to bind to the probe of sequence A1 (Fig. 5D). These results suggested that PpWRKY44 transcriptionally regulates the *PpMYB10* gene by binding directly to the W-box in its promoter.

### PpBBX18 activates the transcription of *PpWRKY44*

**Figure 6:**
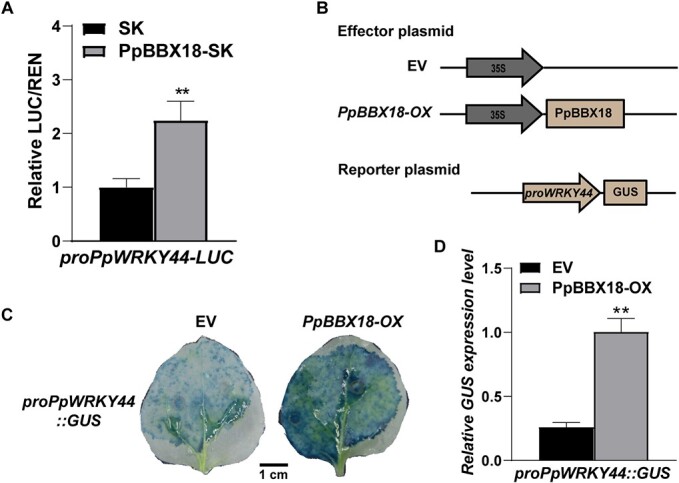
PpBBX18 activates the transcription of *PpWRKY44.* (A) PpBBX18 induced *PpWRKY44* transcription in the dual-luciferase assay. (B) Schematic diagram showing the constructs of effector and reporter used in GUS analysis. (C) Image of GUS staining results for tobacco leaves co-transformed with PpBBX18 and the *PpWRKY44* promoter. Scale bars = 1 cm. (D) Detection of relative *GUS* expression level in tobacco leaves presented in (C) by RT–qPCR. Error bars represent the standard deviation of three biological replicates. ^**^P < .01 (two-tailed Student’s *t*-test).

We previously demonstrated that light-induced PpBBX18 regulates *PpMYB10* transcription, thereby promoting anthocyanin biosynthesis [23]. Interestingly, we note that the *PpWRKY44* expression level significantly increased in the pear calli overexpressing *PpBBX18* (Supplementary Data Fig. S6). To test whether silencing of PpBBX18 also affects the expression of *PpWRKY44*, we performed a transient transformation assay on immature ‘Hongzaosu’ pear fruit with the VIGS vector (TRV2-PpBBX18). As shown in Supplementary Data Fig. S7A and B, silencing of *PpBBX18* suppressed coloration. RT–qPCR analysis revealed that the expression level of *PpWRKY44* was significantly lower in *PpBBX18*-silenced fruit compared with control fruit (Supplementary Data Fig. S7D). Furthermore, the expression levels of *PpMYB10* and anthocyanin-related genes were significantly lower in the *PpBBX18*-silenced fruit compared with control fruit (Supplementary Data Fig. S8). Furthermore, we fused the *PpWRKY44* promoter to a *LUC* reporter gene for a dual-luciferase assay. The observed high luciferase activity implied that PpBBX18 can activate the *PpWRKY44* promoter (Fig. 6A). Moreover, the construct containing the *PpBBX18* coding sequence was co-transformed into the leaves of *N. benthamiana* together with the construct carrying the promoter of *PpWRKY44* fused to the GUS reporter gene (Fig. 6B). We found that coexpressing PpBBX18 with the *PpWRKY44* promoter increased GUS staining (Fig. 6C) and the relative expression level of *GUS* (Fig. 6D). Taken together, these results demonstrate that PpBBX18 stimulates the transcription of *PpWRKY44* to enhance its expression.

## Discussion

### Light-induced PpWRKY44 is a positive regulator of anthocyanin biosynthesis in pear

The WRKY TF superfamily is exclusive to the plant kingdom; its members modulate numerous physiological processes, including flowering, seed and trichome development, and senescence, via indispensable transcriptional regulatory networks [29, 32, 33]. Emerging evidence indicated that WRKY TFs are vital for regulating biotic and abiotic stress responses [24, 43, 44]. There is also convincing evidence that WRKY TFs are implicated in the context of light signaling pathways. In *Arabidopsis*, *AtWRKY22* expression is repressed by light and induced by exposure to darkness [45]. Additionally, AtWRKY63 and AtWRKY40 activate and repress the transcription of genes implicated in the signaling pathway responsive to high-intensity light [46]. In apple, light induces *MdWRKY1* expression, whereas it has the opposite effect on *MdWRKY41* expression [38, 40]. Here we showed that PpWRKY44 is a component of light signaling, with at least three factors that may explain that. First, the expression of *PpWRKY44* was induced when previously bagged pear fruit and pear calli (wild-type) were exposed to light (Fig. 2). Second, the promoter analysis of *PpWRKY44* displayed several light-responsive elements (Supplementary Data Table S1). Third, *PpWRKY44* was activated by PpBBX18, which is one of the light signal transduction pathway components (Fig. 6).

Prior and current studies demonstrated that WRKY TFs negatively or positively affect light-depended anthocyanin biosynthesis in plants [39, 47]. In apple, MdWRKY41 suppressed the expression of *MdMYB12*, *MdANR*, and *MdUFGT*, and negatively regulated the accumulation of anthocyanin in response to light [38]. A recent investigation confirmed that MdWRKY11 enhances fruit coloration by upregulating the transcription levels of their downstream genes *MdMYB10* and structural genes, which are required for anthocyanin biosynthesis in apple [39]. Furthermore, in response to light, apple MdWRKY1 activates *MdLNC499* expression, which leads to upregulated *MdERF109* expression. The generated MdERF109 led to significantly elevated anthocyanin accumulation via binding directly to the *MdbHLH3*, *MdUFGT*, and *MdCHS* promoters [40]. Our previous transcriptomic analysis discovered that the expression levels of more than 25 WRKY-encoding genes in pear are upregulated in response to light. However, their functions in light-responsive processes remain unclear [41]. Similarly, the regulatory functions of WRKY TFs during anthocyanin biosynthesis in red pears have not been thoroughly characterized. In this work, phylogenetic analysis revealed the close relationship between PpWRKY44 and AtWRKY44, which is a Group-I WRKY TF in *Arabidopsis* (Supplementary Data Fig. S1). Moreover, AtWRKY44 influences trichome formation and regulates seed coat tannin production by modulating the vacuolar transport steps in the proanthocyanidin pathway [29, 42]. Furthermore, phenotypic and molecular analyses of the overexpression of *PpWRKY44* in various pear tissues (e.g. leaves and fruit) and in pear calli as well as the effects of silencing *PpWRKY44* in pear fruit indicated that PpWRKY44 positively regulated light-dependent anthocyanin accumulation by increasing the transcription of the regulatory gene *PpMYB10*. These findings prove that *PpWRKY44* is indeed a light-responsive gene, and its expression correlates with light-dependent anthocyanin accumulation in red pears.

### PpWRKY44 promotes anthocyanin biosynthesis by activating *PpMYB10* expression

Light-dependent anthocyanin biosynthesis is indeed a complex process involving the coordinated regulation of several key structural genes, such as *CHS*, *DFR*, and *UFGT* [5, 48, 49], which are mainly transcriptionally regulated by MYB TFs [51]. MYB TFs also act as bridges between specific TFs of environmental signaling components and the anthocyanin structural genes and thus link different signaling pathways before channeling the transcriptional instructions to the structural genes. In this manner, MYB TFs help plants decode environmental cues (e.g. light) into physiological responses (e.g. anthocyanin accumulation) [5, 7, 8, 51]. In apple, MdMYB1 responds to light and affects apple fruit coloration by regulating the transcription of their downstream genes *MdDFR* and *MdUFGT* [12]. Recent studies showed that WRKY TFs regulate the transcription of MYB TF-encoding genes to modulate anthocyanin biosynthesis. For example, anthocyanin production was inhibited by AtWRKY41 in *Arabidopsis* rosette leaves by transcriptionally regulating the three MYB TF genes (*AtMYB75*, *AtMYB111*, and *AtMYBD*) [37]. In apple, proanthocyanidin biosynthesis is inhibited by MdWRKY41, which functions directly upstream of *MdMYB12*, which encodes a positive modulator of proanthocyanidin biosynthesis, to repress its expression [38]. Earlier research indicated that the *MdMYB1* promoter is transcriptionally regulated by MdWRKY72 to promote anthocyanin synthesis in apple [52]. A similar transcriptional regulation was observed in pear. More specifically, in red-skinned pear fruit, PpWRKY26 transcriptionally activates the *PpMYB114* promoter and promotes anthocyanin biosynthesis [53]. The PpMYB10 TF is a critical regulator of anthocyanin biosynthesis in pear because it can directly act upstream of most anthocyanin structural genes [21]. In the current work, PpWRKY44 activated the transcription of *PpMYB10* (Fig. 3). A series of *in vitro* and *in vivo* analyses demonstrated that *PpMYB10* directly acts downstream of PpWRKY44, thereby positively regulating anthocyanin accumulation (Fig. 5).

In plants, anthocyanin biosynthesis is transcriptionally regulated by the MBW complex, which has been widely studied [7, 8]. Recent reports described how WRKY TFs might influence the regulatory effects of the MBW complex [42, 54]. In apple, MdWRKY40 interacts with the vital component of the MBW complex, MdMYB1, to enhance its expression and binding to target genes in response to wounding [47]. Another study revealed that MdWRKY75 stimulates the accumulation of anthocyanins in apples by binding to the promoter of the MYB transcription factor *MdMYB1* and enhancing its activity [55]. PpWRKY26 directly activates *PpMYB114* transcription and interacts with PpbHLH3 to target the *PpMYB114* promoter, ultimately leading to anthocyanin accumulation in red-skinned pear [53]. The novel WRKY–MBW module may be essential for regulating anthocyanin biosynthesis. The results presented herein suggest that PpWRKY44 can positively regulate anthocyanin accumulation via transcriptional regulation of *PpMYB10*, which encodes a key factor of the MYB10–bHLH3–WD40 (i.e. MBW) complex, which regulates anthocyanin biosynthesis in pear.

### PpWRKY44 is part of the light-induced anthocyanin biosynthesis cascade

Recently, BBX proteins have been identified as inducers of anthocyanin biosynthesis in several plants [22, 56]. In a previous study, we revealed that PpBBX18 contributes to the light-induced coloration of pear fruit by regulating the expression of *PpMYB10*, although it cannot bind directly to the *PpMYB10* promoter [23]. Interestingly, we detected a highly significant expression of *PpWRKY44* in calli overexpressing *PpBBX18* (Supplementary Data Fig. S5), suggesting that PpBBX18 might regulate *PpWRKY44* expression. Transient silencing of *PpBBX18* expression in pear fruit confirmed this finding (Supplementary Data Fig. S7). We next performed a dual-luciferase assay and GUS staining. The analysis revealed that PpBBX18 could activate the expression of *PpWRKY44* (Fig. 6). Therefore, in response to light, PpBBX18 may increase the transcription of *PpWRKY44*, which encodes a direct regulator of *PpMYB10* expression. Although further *in vivo* experiments are needed, these results generate the interesting hypothesis that PpWRKY44 might involve the light-induced anthocyanin biosynthesis cascade (PpBBX18–PpWRKY44–PpMYB10) in red pears.

**Figure 7:**
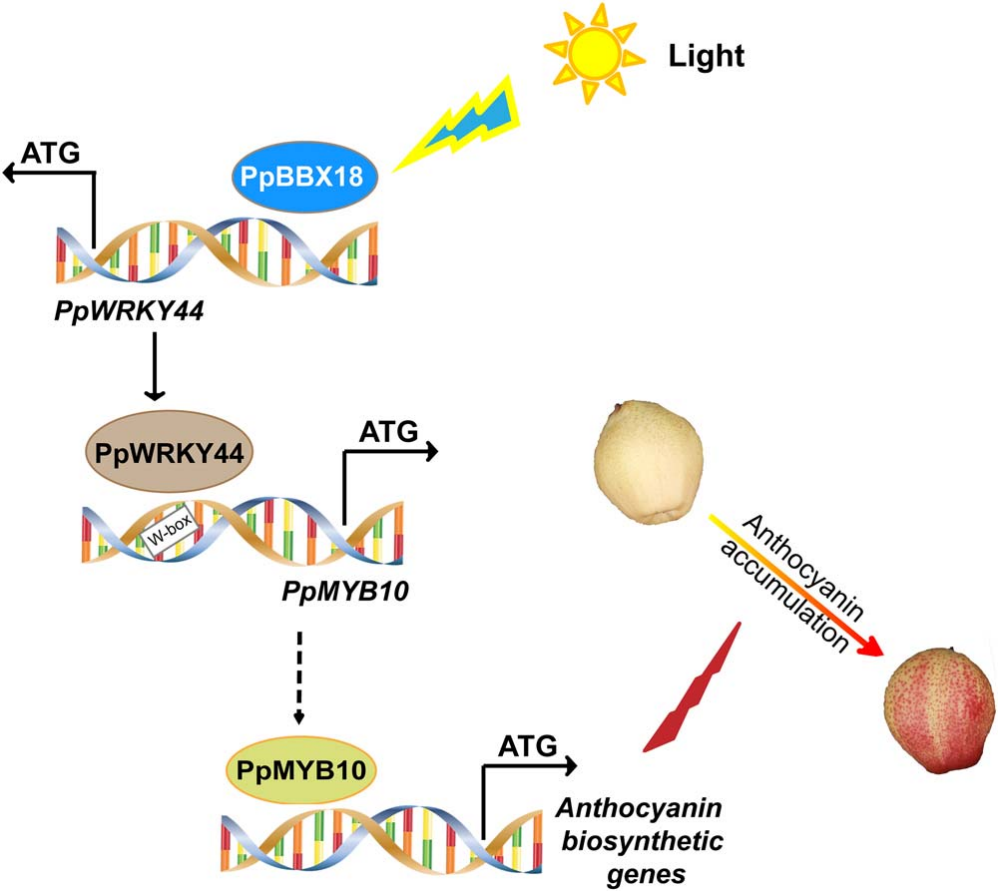
A simplified model for the regulation by PpWRKY44 of light-induced anthocyanin accumulation. Under light, PpBBX18 targets the promoter of *PpWRKY44* to activate its gene expression. PpWRKY44 proteins activate the expression of anthocyanins regulatory gene, upstream of anthocyanins structural genes, *PpMYB10*.

In conclusion, a light-responsive Group-I WRKY TF (PpWRKY44) in ‘Hongzaosu’ pear fruit was identified. In response to light, *PpWRKY44* is highly expressed downstream of PpBBX18. The encoded TF targets *PpMYB10* promoter fragment W3, containing three W-box elements. The EMSA data indicated that TGTCAC is the specific W-box element in fragment W3 that binds to PpWRKY44, leading to transcriptional regulation (Fig. 7). Hence, we have demonstrated that PpWRKY44 positively regulates light-induced anthocyanin biosynthesis through direct activation of the *PpMYB10* promoter in red pear fruit. Our findings have further elucidated the molecular mechanism underlying WRKY-mediated transcriptional regulation of light-induced anthocyanin biosynthesis regulatory genes in red pear fruit.

## Materials and methods

### Identification and phylogenetic analysis of PpWRKY44 TF

Transcriptome sequencing data from our previous studies investigating light-induced anthocyanin accumulation in pear fruit and calli [23, 43] were used to identify the light-induced WRKY TF. The database of The Arabidopsis Information Resource (TAIR, https://www.arabidopsis.org/) was used for *Arabidopsis* WRKY protein sequences. In contrast, the pear WRKY protein sequences from pear genome data were identified using local BLAST analysis. The sequence alignment of pear and *Arabidopsis* WRKY proteins was constructed using ClustalW in MEGA 7.0. IQ-TREE was used for the inferred phylogenetic tree via the maximum likelihood method, with 1000 ultrafast bootstrap replicates [58]. The phylogenetic tree was visualized by using the iTOL program (https://itol.embl.de/). Multiple sequence alignment and characterization of the conserved WRKY domains in the proteins of pear and other species were performed using DNAMAN software.

### Subcellular localization

To determine the subcellular localization of PpWRKY44, the coding sequences of *PpWRKY44* were amplified without a stop codon from skin cDNA prepared from the pear cultivar ‘Hongzaosu’ using primer sequences shown in Supplementary Data Table S2 and fused into the pCAMBIA1300 vector, including the GFP tag sequence. The empty vector of pCAMBIA1300 was employed as control. The constructs were introduced into strain GV3101 of *A. tumefaciens* cells. *Nicotiana benthamiana* (mCherry nuclear expression) leaf infiltration was conducted as described previously [58]. GFP fluorescence in the transiently transformed leaves was analyzed and imaged using the A1 confocal laser scanning microscope (Nikon, Japan).

### Plant materials and light treatments

Bagged pear (*Pyrus pyrifolia* × *P. communis* cultivar ’Hongzaosu’) fruits used in the current study were collected from an orchard 150 days after full bloom. Then, the bagged fruit was quickly taken to the laboratory and maintained in the dark at 22°C overnight. Dedifferentiated pear calli were easy to prepare from the flesh cells of young *P. communis* ‘Clapp’s Favorite’ fruit compared with *P. pyrifolia* and were used in this study. They were cultured on Murashige and Skoog (MS) solid medium containing 30 g l^−1^ sucrose, 1.0 mg l^−1^ 2,4-dichlorophenoxyacetic acid, and 0.5 mg l^−1^ 6-benzylaminopurine at 22°C in the dark. The calli were subcultured before being used for light treatment and genetic transformation three times at 20-day intervals.

For the light response assay, harvested fruits were treated with light as described previously [59]. Briefly, the bagged fruits were separated into two groups and placed in a phytotron at 17°C. One group was exposed to light (60 μmol m^−2^ s^−1^), whereas the fruits in the other group were not exposed to light (i.e. control fruits). After starting the light treatment, the exposed side of the peel of each fruit was scraped at 0, 6, 12, 24, 48, 72, 144, and 240 hours. Three biological replicates were prepared for each sample time-point, with three fruits used for one biological replicate.

For the light response assay, pear calli were treated with light in a phytotron at 17°C. Samples were collected after starting the light treatment at 0, 12, 24, 48, 72, 144, and 240 hours. The pear calli used as controls were covered with aluminum foil. For further analysis, the pear fruit and calli samples were maintained at −80°C after being quickly frozen in liquid nitrogen.

### Genetic transformation

To generate the transgenic pear calli, the constructs 35S:*PpWRKY44–GFP* and 35S:*GFP* (i.e. *GFP* alone) were used. The constructs were separately introduced into strain EHA105 of *A. tumefaciens* cells, followed by transformation into pear calli by means of the *A. tumefaciens*-mediated method as described previously [23]. The transgenic calli were cultured under continuous dark conditions on MS-based solid medium at 22°C. The medium was supplemented with 10 mg/l hygromycin and 200 mg/l timentin. After confirming that they were transformed correctly, the transgenic calli were subcultured onto fresh regeneration medium every 15–20 days. Regarding the light treatment, transgenic pear calli were exposed for 6 days to continuous light.

### Anthocyanin measurements

The contents of anthocyanin in pear peel and calli were measured with slight modifications as described previously [60]. In brief, pear peel and calli were powdered in liquid nitrogen. Then, 0.1 g was weighed and maintained in the dark at 4°C overnight in 1 ml of extraction solution (acetic acid:methanol = 1:99, v/v). The absorbance of each 100-μl sample was measured (at 530, 620, and 650 nm) with a DU800 spectrophotometer (Beckman Coulter, USA). The formula [[(A530 − A650) − 0.2 × (A650 − A620)]/sample quantity] was employed to determine the anthocyanin content.

### RNA extraction, cDNA synthesis, and gene expression analysis

RNAs from the pear peel and pear calli of WT and transgenic lines were isolated based on a modified CTAB method as described previously [61]. First-strand cDNA was synthesized from 1 μg of isolated RNA with the HiScript^®^ II Q RT SuperMix for qPCR (+gDNA wiper; Vazyme Biotech). The generated cDNA was a template for RT–qPCR assays with gene-specific primers (Supplementary Data Table S2) using iTaq™ Universal SYBR^®^ Green Supermix (Bio-Rad, https://www.bio-rad.com/). The 2^−ΔΔCT^ method was utilized to estimate the relative transcription values for RT–qPCR normalization using pear *PpActin* (JN684184) as the reference gene.

### Transient transformation of pear leaves and fruits

A transient gene expression assay was employed to overexpress PpWRKY44 in mature ‘Meirensu’ pear fruit. The coding sequences of *PpWRKY44* were amplified from skin cDNA of ‘Hongzaosu’ using primer sequences shown in Supplementary Data Table S2, and fused into the pGreenII0029 62-SK vector to construct PpWRKY44–SK. After the empty SK and PpWRKY44–SK constructs were delivered into strain GV3101 of *A. tumefaciens* cells, transient overexpression experiments were performed as described previously [23] by means of the GM-0.33A vacuum pump (Jinteng, China). For the pear fruit infiltration, 15 bagged fruits were infiltrated with the EV, while 15 bagged fruits were infiltrated with PpWRKY44-SK (PpWRKY44-OX). VIGS assays were used to silence PpWRKY44 in the ‘Hongzaosu’ pear fruit. A specific 318 bp-long DNA fragment of the coding sequences region of *PpWRKY44* was amplified from skin cDNA of ‘Hongzaosu’ using primer sequences shown in Supplementary Data Table S2, and fused into the pTRV2 vector to construct pTRV2–PpWRKY44. VIGS experiments were performed after the pTRV2–PpWRKY44, pTRV1, and pTRV2 vectors were introduced into strain EHA105 of *A. tumefaciens*, as described previously [23]. For pear fruit injection, 15 bagged fruits were injected with the EV (pTRV1:pTRV2 = 1:1, v/v), while 15 bagged fruits were injected with pTRV2–PpWRKY44 (pTRV1:pTRV2–PpWRKY44 = 1:1, v/v). The ‘Meirensu’-infiltrated fruits and ‘Hongzaosu’-injected fruits were then kept in darkness for 24 hours and then placed in a continuous light incubator for 5 days for gene overexpression assays and 7 days for VIGS assays. After photographing them, fruit peels near the infiltration site were scraped and kept at −80°C after being quickly frozen in liquid nitrogen. For the transient transformation of pear leaves (*Pyrus ussuriensis*), transient pear fruit vectors were also used. The detached leaves were mixed with *A. tumefaciens* cells (GV3101) containing the recombinant vectors and then infiltrated for 20 minutes using a GM-0.33A vacuum pump (Jinteng, China), and placed in darkness for 1 day. After 2 days of light treatment, pear leaves were photographed and sampled to extract RNA and measure anthocyanin content.

### Dual-luciferase assay

The transient expression assay followed the protocol described previously [62]. In brief, the coding sequences of *PpWRKY44* were amplified from skin cDNA of ‘Hongzaosu’ and ligated with the pGreenII0029 62–SK vector, creating the effector construct. The promoter of *PpMYB10* was cloned from genomic DNA of ‘Hongzaosu’ into the pGreenII0800–LUC vector, creating the reporter construct. Both constructs were separately delivered into strain GV3101 of *A. tumefaciens* cells. *Agrobacterium* strains containing recombinant constructs were combined at a volume ratio of 10:1 (10 PpWRKY44–SK, 1 ProPpMYB10–LUC) before co-transformation into *N. benthamiana* leaves. For the negative control, the leaves were injected with a combination of cells containing pGreenII0029 62–SK and ProPpMYB10–LUC. *Renilla* and firefly luciferase activities were tested 2.5 days after injection by means of a Dual-Luciferase Reporter Assay Kit (Promega, https://www.promega.com) based on the operating instructions. Primers provided in Supplementary Data Table S2 were utilized to amplify the promoter of *PpMYB10* and the coding sequence of PpWRKY44.

### Yeast one-hybrid assays

According to the Yeast Protocols Handbook (Clontech), a Y1H assay was performed. The *PpMYB10* promoter fragments were amplified from genomic DNA of ‘Hongzaosu’ by means of the primers provided in Supplementary Data Table S2, and incorporated into the pAbAi vector. The vector was then sequenced and inserted into Y1HGold yeast cells. Y1HGold cells harboring the *PpMYB10*–pAbAi vector were added to SD/−Ura plates to test promoter auto-activation and select positive colonies. The coding sequences of *PpWRKY44* were ligated with the pGADT7 prey vector (AD). The Y1HGold strain harboring the *PpMYB10*–pAbAi vector was re-transformed with PpWRKY44–AD or the empty AD plasmid. Positive interactions were selected at 30°C for 5 days on SD/−Leu plates containing aureobasidin A (AbA).

### Chromatin immunoprecipitation–qPCR assays

The ChIP–qPCR assays were conducted as described previously [63]. Light-treated transgenic pear calli containing PpWRKY44–GFP or GFP alone were collected for subsequent cross-linking with formaldehyde (1%) under vacuum conditions for 15 minutes. Cross-linking was stopped by adding glycine (125 mM final concentration) and maintaining vacuum conditions for 10 minutes. The chromatin DNA was then extracted via sucrose gradient centrifugation, and sonicated at 4°C for 30 minutes (30 seconds with 30-second intervals) using the Bioruptor Plus device (Diagenode) to produce 200- to 300-bp random fragments. The sonicated chromatin was immunoprecipitated overnight using anti-GFP antibodies (Abcam, China), after which qPCR analysis was used to determine the amount of immunoprecipitated chromatin.

### Electrophoretic mobility shift assay

The coding sequences of *PpWRKY44* were amplified from skin cDNA of ‘Hongzaosu’ using the primers provided in Supplementary Data Table S2. It was then ligated with the pET-32a vector containing a His tag using BamHI and HindIII restriction enzymes. For protein induction, recombinant vector was introduced into strain BL21 *Escherichia coli* cells and the cells were incubated overnight at 16°C with 0.2 mM isopropyl-β-d-thiogalactopyranoside. The fusion protein was purified utilizing Ni-NTA Sefinose™ Resin (Sangon Biotech, China). For preparing the probes, probes labeled with biotin at the 3′ end were synthesized (Genebio, China), followed by the preparation of double-stranded DNA probes as described previously [64]. An EMSA was performed using a LightShift™ Chemiluminescent EMSA Kit (Thermo Fisher Scientific, USA). Briefly, purified recombinant His-PpWRKY44 was incubated with biotin-labeled probes for 30 minutes at room temperature. Then, the reaction mixture was separated by PAGE at 200 V, transferred to a nylon membrane (Millipore, http://www.merckmillipore.com/), and subjected to UV cross-linking. Finally, anti-biotin antibody was used to detect the biotin-labeled probes.

### GUS staining assays

The promoter of *PpWRKY44* (~1500 bp) was cloned from genomic DNA of ‘Hongzaosu’ into the pCAMBIA1301 vector upstream of the *GUS* gene, creating the reporter vector. The coding sequences of *PpBBX18* were amplified from skin cDNA of ‘Hongzaosu’ and inserted into pCAMBIA1300–GFP, creating the effector vector. *Agrobacterium* (*A. tumefaciens* GV3101-pSoup) cells containing the reporter and effector vectors, after mixing equally (v/v), were transiently expressed in *N. benthamiana* using 4-week-old tobacco plants with three leaves as described previously [63]. For GUS staining, the leaves were dipped in GUS staining solution, infiltrated for 15 minutes using a GM-0.33A vacuum pump (Jinteng, China), placed in darkness overnight at 37°C, and then quickly frozen in liquid nitrogen to analyze the *GUS* expression level by RT–qPCR according to a previously described method [65]. The other leaves were washed in ethanol (80%) to remove chlorophyll before photographing.

### Statistical analysis

Samples were statistically analyzed with two-tailed Student’s *t*-test using GraphPad Prism version 8.0.

## Acknowledgements

This work was supported by the National Key Research and Development Program (2018YFD1000200), the China Agriculture Research System of MOF and MARA and the Fundamental Research Funds for the Central Universities (2021QNA6022).

## Author contributions

Y.T. and S.B. planned and designed the research; A.A. conducted most of the experiments with help from X.Z., L.P., and L.Z.; Y.G. contributed to the bioinformatics analysis; M.A. and J.N. provided help and advice; A.A., M.A., J.N., S.B., and Y.T. wrote the manuscript. All authors read and approved the manuscript for submission.

## Data availability

All relevant data in this study are provided in the article and its supplementary files.

## Conflicts of interest

The authors declare no competing interests.

## Supplementary Data

Supplementary data is available at *Horticulture Research* online.

## Supplementary Material

Web_Material_uhac199
