# Supplementary material for: Light-responsive transcription factor PpWRKY44 induces anthocyanin accumulation by regulating PpMYB10 expression in pear
Source: Hortic Res. 2022 Sep 6;9:uhac199. doi: 10.1093/hr/uhac199 (PMC10167416; doi:10.1093/hr/uhac199)

**Supplementary Tables:**

**Table S1. The promoter analysis of PpWRKY44 indicated the light-responsive elements predicted by the PlantCARE database.**

| Motif | Strand | Distance from ATG | Sequance | Putative function |
| --- | --- | --- | --- | --- |
| TCT-motif | + | 1386 | TCTTAC | part of a light responsive element |
| ATC-motif | + | 604 | TGCTATCCA | part of a conserved DNA module involved in light responsiveness |
| G-box | - | 1869 | CACGTG | cis-acting regulatory element involved in light responsiveness |
| Box4 | + | 238 | ATTAAT | part of a conserved DNA module involved in light responsiveness |
|  | - | 1193 | ATTAAT |  |
| Chs-Unit1m1 | + | 538 | ACCTAACCCGC | part of a light responsive element |
| Gap-box | + | 1176 | CAAATGAA(A/G)A | part of a light responsive element |
| GATA-motif | + | 1301 | GATAGGA | part of a light responsive element |
| MRE | + | 1333 | AACCTAA | MYB binding site involved in light responsiveness |

**Table S2. Primers used in the study.**

| Primer name | Sequance (5’ to 3’) |
| --- | --- |
| PpWRKY44-1300-F | ATGGATATTAAGGAAGCAGAGC |
| PpWRKY44-1300-R | TCATGGCTTTTCTTTAGTTGT |
| PpWRKY44 (qRT)-F | TGGACTGCCGGTGCATTTTT |
| PpWRKY44 (qRT)-R | TCAAGCCCCTTGCTTCCTTC |
| PpMYB10 (qRT)-F | CAGCAGAAGATTTAAGTACGCCATC |
| PpMYB10 (qRT)-R | TTCTAACAAGGTCTCCCACCAATC |
| PpWRKY44-SK-F | ATGGATATTAAGGAAGCAGAGC |
| PpWRKY44-SK-R | TCATGGCTTTTCTTTAGTTGT |
| PpMYB10-LUC-F | GGATATAAACCACTCAACGAAG |
| PpMYB10-LUC-R | CTCGTATCTGCTAGCAGCTAAGC |
| PpWRKY44-AD-F | ATGGATATTAAGGAAGCAGAGC |
| PpWRKY44-AD-R | TCATGGCTTTTCTTTAGTTGT |
| PpMYB10-pAbai-F1 | TACGGGCTAGGATTCTCTCC |
| PpMYB10-pAbai-R1 | CTCGTATCTGCTAGCAGCTAAG |
| PpMYB10-pAbai-F2 | GGATATAAACCACTCAACGAAG |
| PpMYB10-pAbai-R2 | GTCACATCAACATCTTATATTAAT |
| PpWRKY44-pET-32a-F | ATGGATATTAAGGAAGCAGAGC |
| PpWRKY44- pET-32a-R | TCATGGCTTTTCTTTAGTTGT |
| ChIP-ProMYB10-F1 | GAAGAGATTTCTACTTTATA |
| ChIP-ProMYB10-R1 | GTGAAGGCAGAATCGTGTAAC |
| ChIP-ProMYB10-F2 | GTTACACGATTCTGCCTTCAC |
| ChIP-ProMYB10-R2 | GCTACCAGTCTAACTTGTGA |
| ChIP-ProMYB10-F3 | TCACAAGTTAGACTGGTAGC |
| ChIP-ProMYB10-R3 | TTTGCCTGCTACCCACTTCA |
| PpWRKY44-pTRV2-F | GCCTCCGAAAATAGCCTCGC |
| PpWRKY44-pTRV2-R | CCAGTTCCATCAGAGGCTA |
| PpCHI (qRT)-F | GAACGGGTGCAAGGAATCTA |
| PpCHI (qRT)-R | AACAGGAGTCCCTCCCAAGT |
| PpCHS (qRT)-F | GGGTGTACTCTTCGGATTTGG |
| PpCHS (qRT)-R | TGAAGTTGAATGGAATGGAATGC |
| PpDFR (qRT)-F | ACTGAGGCTGCTGAGGAGAG |
| PpDFR (qRT)-R | TCAAATCCAAGCTGGTAAATGT |
| PpANS (qRT)-F | AGTTGTTCAGGAAAAGCCAAGAGG |
| PpANS (qRT)-R | ACAAAGCAGGCAGATAGGAGTAGC |
| PpF3H (qRT)-F | GGAGAAAGACAAAGTGGAGATAAAGC |
| PpF3H (qRT)-R | ACAAGAAGTGGAAAGGCAAAGTTAC |
| PpUFGT (qRT)-F | CTGGAACCTGAAGTTGTGAATCTG |
| PpUFGT (qRT)-R | AGCCACTCTAAGCAACCACTATC |
| PpActin (qRT)-F | CCATCCAGGCTGTTCTCTC |
| PpActin (qRT)-R | GCAAGGTCCAGACGAAGG |

**Supplementary Figures:**

**Figure S1. RPKM value of PpWRKY44 in pear calli after two days under light and dark treatment.**

RNA-SEQ data was reported previously (Bai et al., 2017). Statistics were determined using two-tailed Student’s *t*-test (^*^*P*< 0.05, ^**^*P*< 0.01).

**
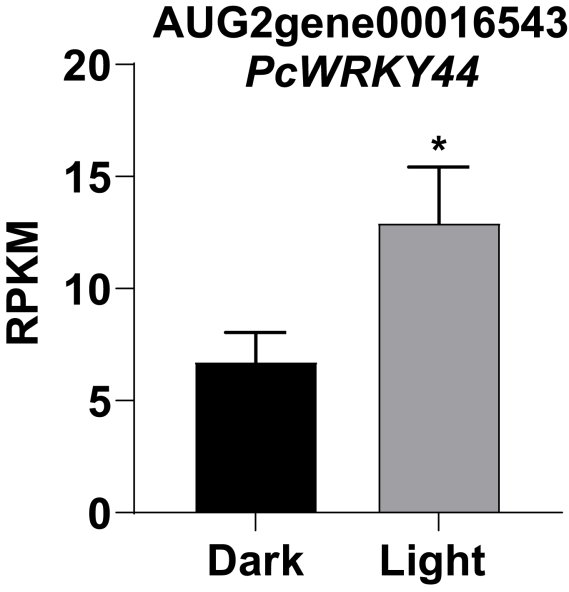
**

**Figure S2**. **A phylogenetic tree of some WRKY proteins from pear and Arabidopsis.**

Accession numbers in red refer to the proteins of pear and in black refer to the proteins Arabidopsis. A yellow background distinguishes PpWRKY44 (Pbr008278.1) and AtTTG2.


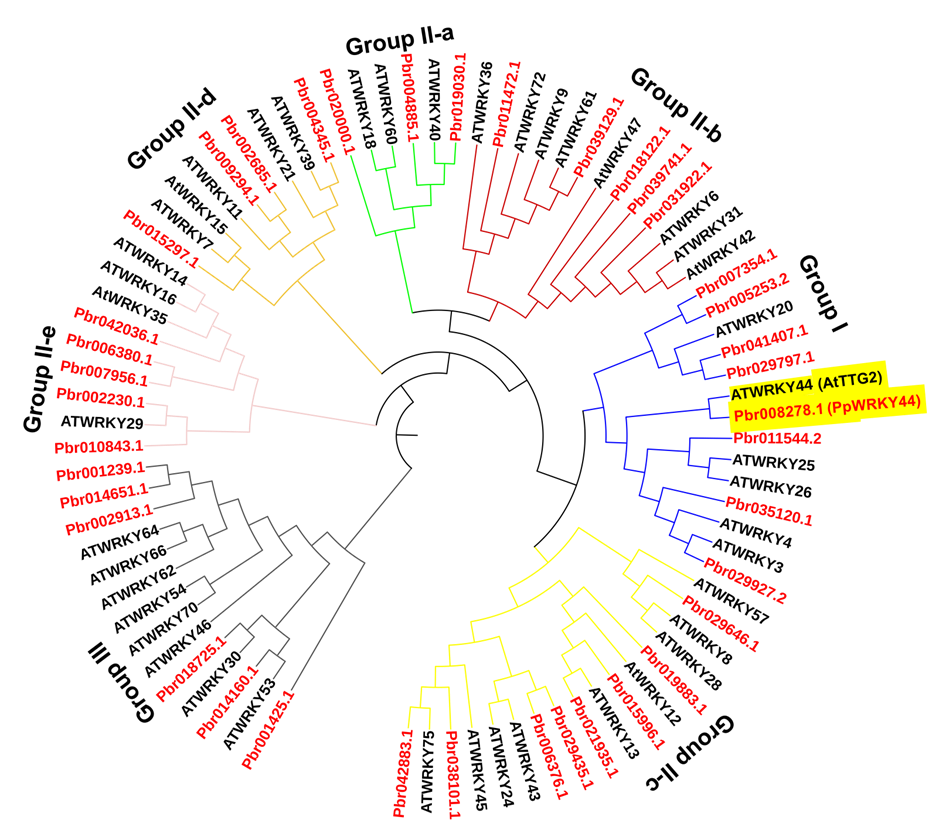


**Figure S3. Expression level patterns of anthocyanin biosynthetic genes in light-treated ‘Hongzaosu’ pear fruit.**

RT-qPCR analyses showing the relative expression levels of pear anthocyanin biosynthetic genes (A-I), *PpBBX18* (A), *PpMYB10* (B), *PpMYB114* (C), *PpCHS* (D), *PpCHI* (E), *PpF3H* (F), *PpDFR* (G), *PpANS* (H) and *PpUFGT* (I) in light-treated ‘Hongzaosu’ pear fruit (Fig. 1). The expression level of 0 h was used as the reference. Error bars represent the standard deviation of three biological replicates. Statistics were determined using two-tailed Student’s t-test *(*P* < 0.05*, **P* < 0.01).

**
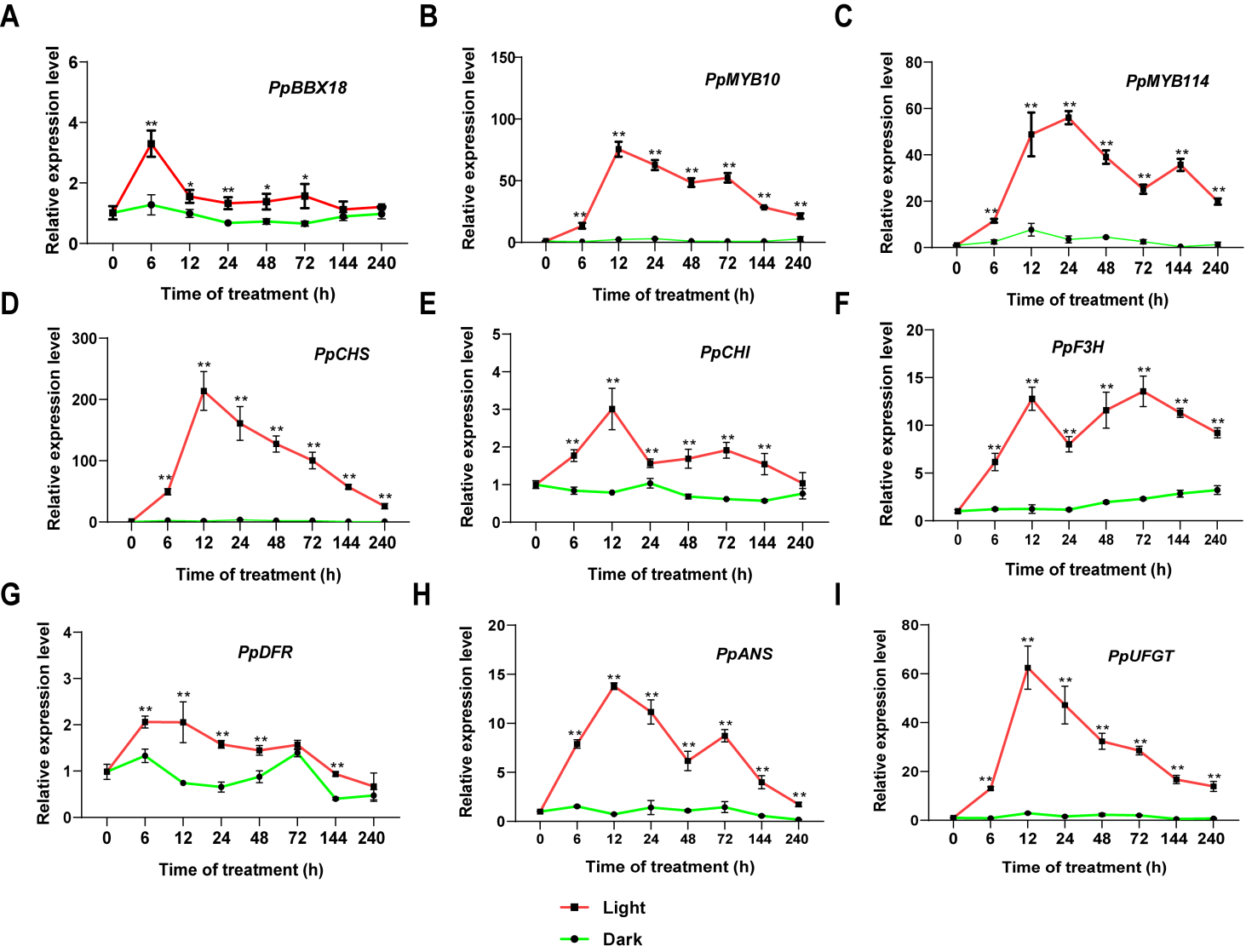
**

**Figure S4. Validation of transgenic calli based on a reverse transcription PCR analysis.**

The coding sequences of *PpWRKY44* were amplified from template cDNA prepared from the pear cultivar ‘Hongzaosu’ and inserted into the pCAMBIA1300 vector, including the GFP tag sequence, to produce transgenic calli. The GFP vector-specific primers were used for the PCR amplification. Lane M, DNA Marker DL5,000; R, replications.

**
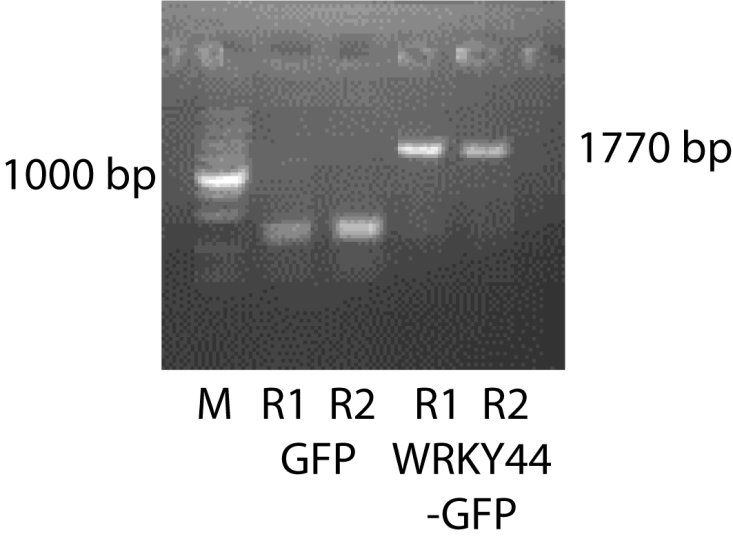
**

**Figure S5**. **PpWRKY44 activated the promoters of anthocyanin biosynthesis-associated genes according to dual-luciferase assays.** PpWRKY44 activated the transcription of the promoters of *PpCHI*, *PpANS*, *PpDFR* and *PpUFGT*, but not the *PpCHS* promoter. Error bars represent the standard deviation of three biological replicates. Statistics were determined using two-tailed Student’s t-test *(*P* < 0.05*, **P* < 0.01).


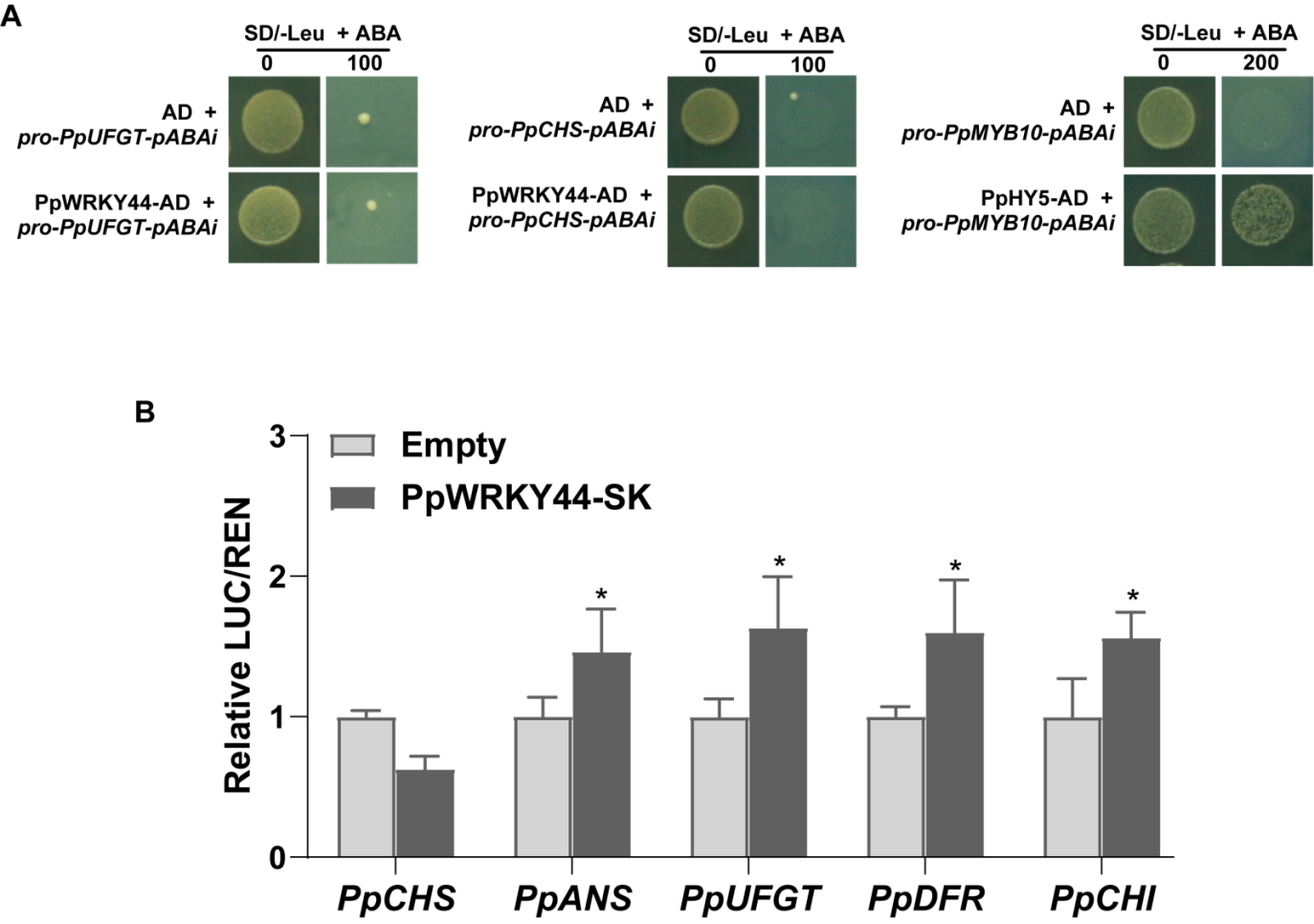


**Figure S6. PpBBX18 activates the *PpWRKY44* expression*.***


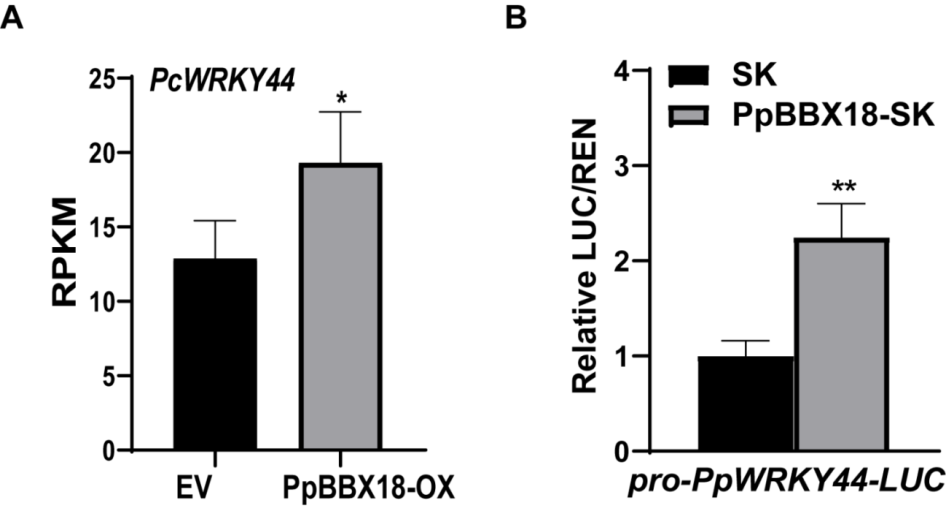
The RPKM values of *PcWRKY44* were calculated based on transcriptome data for *PpBBX18*-overexpressing pear calli under the light. RNA-SEQ data was reported previously (Bai et al., 2019*b*). Error bars represent the standard deviation of three biological replicates. Statistics were determined using two-tailed Student’s *t*-test (^*^*P*< 0.05, ^**^*P*< 0.01).

**Figure S7. Transient silencing of *PpBBX18* expression in pear fruit.**

**A** Transient silencing of *PpBBX18* reduced the accumulation of anthocyanin in immature ‘Hongzaosu’ fruit after a 6-day light treatment. Bars: 1 cm. **B** Anthocyanin contents around the infiltrated sites of the pear fruit in which *PpBBX18* was transiently silenced. **C** *PpBBX18* expression in the pear fruit in which *PpBBX18* was transiently silenced. **D** Relative *PpWRKY44* expression level in the pear fruit in which *PpBBX18* was transiently silenced. Error bars represent the standard deviation of three biological replicates. The expression level of EV was used as the reference. Statistics were determined using two-tailed Student’s *t*-test (^*^*P*< 0.05, ^**^*P*< 0.01).

**
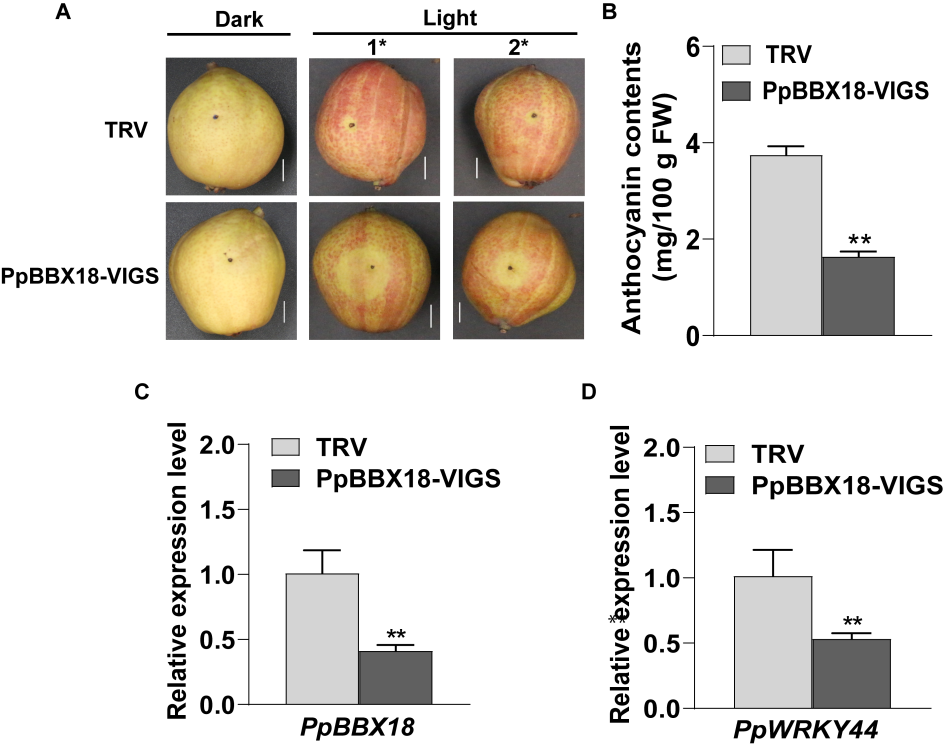
**

**Figure S8. The expression levels of *PpMYB10* and anthocyanin biosynthesis-related genes in the transient silencing of *PpBBX18* in pear fruit (Fig. S7).**

Error bars represent the standard deviation of three biological replicates. The expression level of EV was used as the reference. Statistics were determined using two-tailed Student’s *t*-test (^*^*P*< 0.05, ^**^*P*< 0.01).


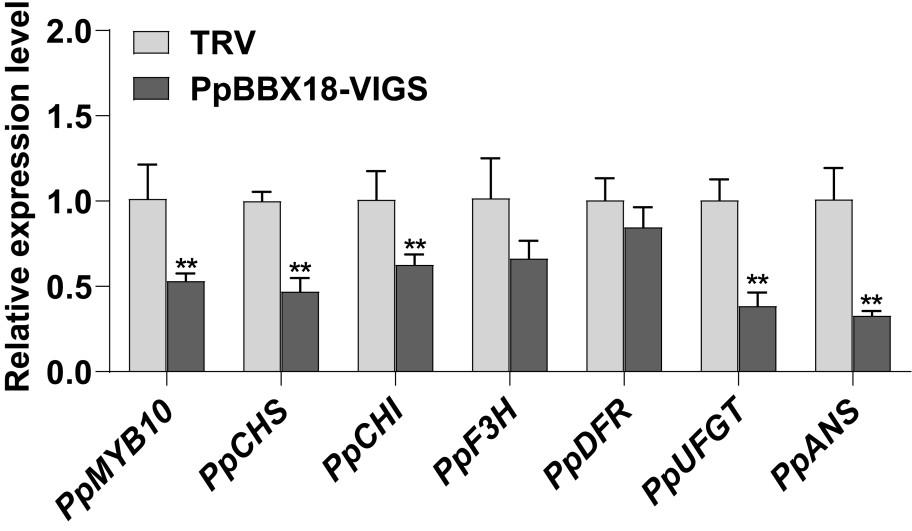

Supplement: Web_Material_uhac199 [file web_material_uhac199.docx]
